# Influenza Outbreak in a Vaccinated Population — USS Ardent, February 2014

**Published:** 2014-10-24

**Authors:** Theodore L. Aquino, Gary T. Brice, Sherry Hayes, Christopher A. Myers, Jaqueline McDowell, Brenda White, Rebecca Garten, Daniel Johnston

**Affiliations:** 1 Mine Counter Measures Squadron Three, U.S. Navy; 2 Naval Health Research Center, U.S. Navy; 3 Navy Environmental and Preventive Medicine Unit Five, U.S. Navy; 4 World Health Organization Collaborating Center for Influenza, CDC; 5 Independent Duty Corpsman, USS Ardent

On February 10, 2014, the USS Ardent, a U.S. Navy minesweeper, was moored in San Diego, California, while conducting training. Over the course of 3 days, 25 of 102 crew members sought medical care because of influenza-like illness (ILI). Nasal swab specimens were collected from each patient, and initial rapid influenza testing indicated 16 cases of influenza A. Ultimately, polymerase chain reaction (PCR) testing conducted by the Naval Health Research Center determined that 20 specimens were influenza A, of which 18 were subtype H3N2. Two specimens could not be subtyped. The HA gene sequence of an outbreak isolate was 99% identical to strains circulating during the 2013–14 influenza season and antigenically similar to the H3N2 component of the 2013–14 influenza vaccine. At the time of the outbreak, 99% of the crew had received influenza vaccine. Through the duration of the outbreak, the minesweeper squadron medical officer collaborated with Navy Environmental and Preventive Medicine Unit Five, higher-level Navy authorities, and County of San Diego Public Health Services to implement the outbreak response, which included disseminating outbreak information to surrounding Navy units, disinfecting the ship, sending home infected crew members, identifying family members at high risk, and providing antiviral medications and guidance. No crew member had onset of symptoms >6 days after the first crew member became ill. This outbreak highlights the risk for an H3N2 influenza outbreak among vaccinated and otherwise healthy young persons.

ILI was defined as illness with two or more of the following symptoms: fever >100.4°F (>38.0°C), chills, sore throat, cough, shortness of breath, congestion, headache, body aches, and nausea. Twenty crew members reported sick on February 10, one on February 11 and four more on February 12. Symptom onset dates were February 5–11 (Figure). All ILI patients were interviewed and examined aboard ship by both an independent duty corpsman (i.e., shipboard medical provider) and a physician. Two nasal swab specimens were taken from each ILI patient by staff members from the Naval Health Research Center. Nasal swab specimens and influenza A and B rapid influenza tests were used for immediate influenza testing. The remaining nasal swab specimens were screened by the Naval Health Research Center for influenza A and B using the CDC PCR assay (1), and DNA sequencing of the HA1 portion of the hemagglutinin gene was performed as previously described (2). Data on demographics and symptomatology were collected using questionnaires and personal interviews.

All 25 crew members with ILI symptoms were otherwise healthy men aged 21–44 years. ILI cases occurred in all ranks, departments, job types, and work shifts. The ship had been in port since being transported from Bahrain to San Diego 2 months before the outbreak. No sailors reported any recent travel. Rapid influenza testing indicated 16 cases of influenza A and nine negative results. Nasal swab specimens from 20 of the 25 ILI patients were positive by PCR for influenza A, with 18 specimens confirmed as A (H3) and two as A (untyped). Influenza A virus was isolated from seven of 11 nasal swab specimens selected for viral culture. These seven specimens had HA1 protein sequences that were identical to each other and differed from the 2013–14 influenza A (H3N2) A/Texas/50/2012 vaccine strain by 5 amino acid substitutions (N128A, R142G, N145S, P198S, and V347K). Sequence analysis (3) of the HA1 portion of the hemagglutinin gene showed 99% homology to typical H3N2 strains circulating in the United States and worldwide during the 2013–14 northern hemisphere influenza season and were found to be antigenically similar to A/Texas/50/2012 (4). Ninety-nine of 102 USS Ardent crew members, 24 of the 25 with ILI symptoms, and 17 of 18 crew members with confirmed influenza A (H3N2) infection had received the 2013–14 influenza vaccine ≥3 months before the outbreak. Vaccinations had been administered at local naval health clinics and at a vaccination fair conducted by Naval Medical Center San Diego. Of the 25 crew members with ILI symptoms, 16 were vaccinated via intradermal injection, eight via intranasal mist, and one had not received vaccination.

Interviews revealed a possible source of the outbreak to be an Ardent crew member (patient A), aged 26 years, who had been evaluated at a local emergency room for fever and cough on January 30, 11 days before the first ILI case was diagnosed. A chest radiograph and computed tomographic scan were performed because of suspicion of pulmonary embolism; both were negative. The patient had been receiving treatment for pyelonephritis, and the clinical impression was that the cough was related to the pyelonephritis. No testing for influenza was performed, and the patient was discharged. Patient A’s roommate in a shore apartment, also a USS Ardent sailor, experienced ILI symptoms on February 5. Because patient A’s roommate was the first of the 25 crew members to experience ILI, and no other probable cause for the outbreak was found, it is possible that patient A actually had influenza. Since patient A did not board USS Ardent because he was ill, it is likely he infected his roommate, who then spread influenza to other USS Ardent crew members.

In an effort to reduce spread and impact of disease, oseltamivir (75 mg twice a day for 5 days) was prescribed to each ILI patient who reported that symptoms had developed within 48 hours of their medical visit, regardless of their vaccination status and rapid influenza testing results. In addition to antiviral medication, rapid identification of the influenza outbreak, and immediate isolation of affected persons (crew members with ILI symptoms were sent off ship to their homes for 48 hours), additional steps to control the outbreak were taken: thorough cleaning of spaces throughout the ship by the crew and use of the ship’s public address system to instruct personnel to wash hands frequently, use hand sanitizer, cover their mouths when coughing, and report for medical evaluation if they were experiencing ILI symptoms. Similar announcements were made aboard three other minesweepers sharing the same pier as USS Ardent. Following a policy implemented by the independent duty corpsman, all patients experiencing ILI symptoms were required to wear an N95 filtering facepiece respirator while shipboard until 5 days after onset of symptoms. Cleaning of spaces was done by regularly disinfecting all commonly touched surfaces with disinfecting wipes and mopping all decks with an iodophor disinfectant diluted to 150 ppm of iodine. E-mails and reports regarding the outbreak, with an emphasis on rapidly identifying patients with ILI, were distributed to all ships on Naval Base San Diego and to high-level Navy officials and County of San Diego Public Health Services. No additional cases were identified after February 14. A total of 43 working days were lost by the 25 ILI patients.

What is already known on this topic?

The single best way to prevent influenza infection is to receive vaccination every year. Some organizations have a mandatory vaccination policy. Despite this, influenza outbreaks can occur in highly vaccinated populations, especially in confined settings.

What is added by this report?

In February 2014, a total of 25 of the 102 crew members of a U.S. Navy minesweeper sought medical care because of influenza-like illness attributed to an influenza A (H3N2) virus antigenically similar to the H3N2 component of the 2013–14 vaccine. Among the crew members, 99% had received influenza vaccination, including 24 of 25 ill persons. Outbreak management included use of an antiviral medication, exclusion of the ill from the ship for 48 hours, disinfection, hand washing, and cough etiquette. No crew member had onset of symptoms >6 days after the first crew member had symptoms.

What are the implications for public health practice?

This influenza outbreak highlights the risk for an outbreak of influenza A (H3N2) in a cohort of vaccinated and otherwise healthy young persons.

## Discussion

USS Ardent, an Avenger class minesweeper, is one of the smallest ships in the U.S. Navy. It has one shared space in which the entire crew eats meals. Work areas are spread throughout the ship, and there are nine sleeping spaces. Military populations, especially those living and working in confined settings, are susceptible to respiratory disease outbreaks (5). Shipboard personnel are at especially high risk because of constant close quarter exposure to a large number of crew members (6). Virtually all areas onboard ships are shared, and movement frequently requires touching handrails, door knobs, and other objects that can be contaminated with nasal secretions. In addition, ventilation systems can circulate infectious pathogens throughout a ship (7).

As the ship was moored in San Diego, the entire crew worked onboard during the day, and 25% remained onboard through each night. The roster of crew members who remained onboard at night rotated daily. There were 16 cases of confirmed influenza A (H3N2)infection in San Diego County (Brit H. Colanter, MPH, Health and Human Services Agency County of San Diego, personal communication, 2014) during the 6 weeks leading to the ship outbreak, making it likely that the virus was acquired from the local community.

Since the 1950s, a policy of mandatory annual vaccination against influenza for active duty personnel has been largely successful in limiting influenza epidemics in the military (8). The current U.S. Department of Defense influenza vaccination policy mandates that all uniformed personnel receive seasonal influenza vaccination, unless medically exempt, or face punishment under the Uniform Code of Military Justice. The policy specifically directs all Navy operational units to be at least 90% vaccinated. However, despite vaccination measures, influenza outbreaks can still occur in highly vaccinated military populations (9,10).

## Figures and Tables

**FIGURE:**
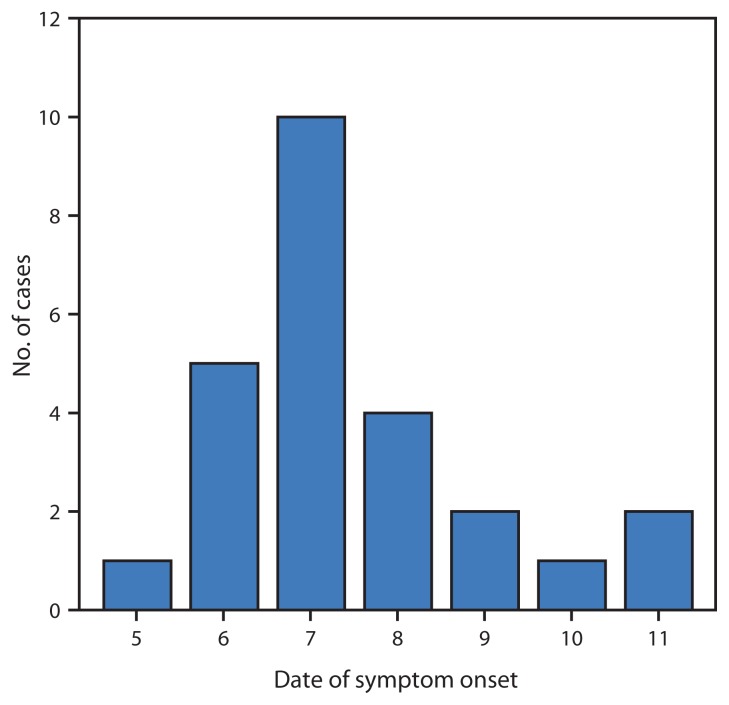
Number of cases (N = 25) of influenza-like illness, by date of symptom onset — USS Ardent, February 5–11, 2014
